# Quantized angular momentum in topological optical systems

**DOI:** 10.1038/s41467-018-08215-5

**Published:** 2019-01-21

**Authors:** Mário G. Silveirinha

**Affiliations:** University of Lisbon–Instituto Superior Técnico and Instituto de Telecomunicações, Avenida Rovisco Pais, 1, 1049-001 Lisboa, Portugal

## Abstract

The Chern index characterizes the topological phases of nonreciprocal photonic systems. Unlike in electronics, the photonic Chern number has no clear physical meaning, except that it determines the number of unidirectional edge states supported by an interface with a trivial mirror. Here, we fill in this gap by demonstrating that the photonic Chern number can be understood as the quantum of the light-angular momentum in a photonic insulator cavity. It is proven that for a large cavity, the thermal fluctuation-induced angular momentum is precisely quantized in the band-gaps of the bulk states. The nontrivial expectation of the light angular momentum is due to a circulation of thermal energy in closed orbits. Remarkably, this result can be extended to systems without a topological classification, and in such a case the “quantum” of the angular momentum density is determined by the net number of unidirectional edge states supported by the cavity walls.

## Abstract (web summary)

While the Chern number is used to characterize topological states in both electronic and photonic systems, its direct physical meaning in photonics has not yet been established. Here, Silveirinha shows that the photonic Chern number can be understood as a quantum of the optical angular momentum.

## Introduction

Topological matter and topological effects have elicited a great deal of interest in recent years, first in electronics^1–5^ and then in photonics and acoustics^6–13^. Notably, topological materials may enable the propagation of waves totally immune to the back-scattering due to either deformations of the propagation path, sharp corners, or due to defects. Hence, topological systems may enable a more efficient transport of light and avoid unwanted feedback and loss due to back-reflections^6–13^. There are several sub-classes of photonic materials with topological properties^9–11^, but only optical systems with a broken time-reversal symmetry provide a strong topological protection^14,15^. In such systems, the different topological phases are characterized by a topological index known as the Chern number.

In the fermionic case the Chern number has an immediate physical meaning: it determines the value of the quantized Hall conductivity of a 2D electron gas^4,16^, and thus the electronic transport properties in the zero temperature limit. In contrast, in photonics the Chern number has not been linked with any specific physical quantity, except that similar to electronics it determines the number of gapless unidirectional edge states at an interface with a trivial insulator^6,8,14,15^. In this article, we fill in this gap of understanding by proving that the (thermal or quantum) fluctuation-induced light angular momentum in a generic photonic-insulator cavity in thermodynamic equilibrium with a large reservoir has a spectral density per unit of area that is quantized in units of $\frac{1}{{\pi c^2}}{\cal{E}}_{{{T}},\omega }$, with ${\cal{E}} _{{{T}},\omega } = \frac{{{\hbar} \omega }}{2}{\mathrm{coth}}\left( {\frac{{{\hbar} \omega }}{{2k_{\mathrm{B}}{{T}}}}} \right)$ the mean energy of a harmonic oscillator at temperature *T*^17^. The nontrivial light angular momentum is due to a circulation of “heat” in closed orbits^18^. This result is rather general and remarkably applies even to systems without any topological classification. In the case of topological systems, the quantized angular momentum spectral density is precisely determined by the photonic Chern number.

## Results

### The topological cavity

**Fig. 1:**
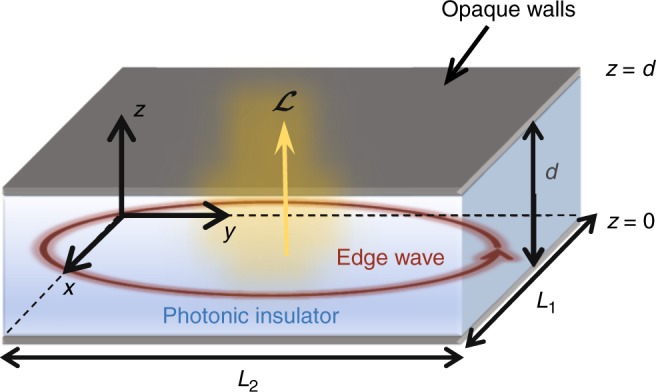
Geometry of a generic “photonic insulator” cavity. The closed electromagnetic cavity does not support bulk states in some spectral range (“photonic insulator”). The lateral walls (not shown) may eventually enable the propagation of edge-type waves confined to the boundary. The thermal fluctuation-induced light is generally characterized by a non-trivial angular momentum ${\cal L}$, which for a sufficiently large cavity has a quantized spectral density in a band-gap

We consider a generic closed cavity filled with either a photonic crystal or, alternatively, an electromagnetic continuum with no intrinsic periodicity (Fig. 1). The cavity cross-section shape in the *xoy*-plane can be rather arbitrary, but for simplicity, we focus on a rectangular cross-section with dimensions *L*_1_ × *L*_2_. The cavity’s height along the *z*-direction is *d*. The cavity walls are assumed “opaque”, i.e., impenetrable by light. For example, perfectly electric conducting (PEC) walls are opaque. The cavity may be regarded as a parallel-plate waveguide (with propagation plane parallel to *xoy*) terminated with the lateral walls. The equivalent unbounded waveguide does not support electromagnetic states in the spectral range of interest (photonic insulator). Note that the band structure is determined not only by the materials inside the cavity but also by the top and bottom walls. For simplicity, the effects of material loss in the wave propagation are assumed negligible. Recently, it was shown that topological concepts may be extended to some non-Hermitian systems, e.g., systems with loss and or gain^19–23^.

Importantly, even when there are no bulk states, the opaque boundaries may enable the propagation of edge states localized at the cavity lateral walls^18,24^. Such edge states famously occur in topological platforms^6,7,9,14,15^, but they may as well emerge in systems with no topological classification^25^. Furthermore, the edge-states may be supported even when the system is time-reversal invariant (reciprocal)^9,24,25^.

### Angular momentum of an edge state

Consider a generic edge-state circulating around the cavity lateral walls (Fig. 1). The circulating motion is evidently associated with a non-trivial light angular momentum given by ${\cal L} = \frac{1}{{c^2}}{\int} {{\mathrm {d}}V} \,{\mathbf{r}} \times {\mathbf{S}}$, where **S** is the Poynting vector, **r** = (*x, y, z*) is a generic point in space, and the integration is over the cavity volume. The angular momentum of photonic systems is extensively discussed in refs. ^26–29^. With reference to the nomenclature of ref. ^29^, we adopt a “kinetic picture” and the Abraham formalism so that the electromagnetic momentum density is **S**/*c*^2^ ^30–32^. In general, the angular momentum may be decomposed into orbital and spin components^29^. The spin component is origin independent, but for an open system the orbital component (and thereby also the total angular momentum) generally depends on the origin of the coordinate axes^29^. In contrast, for a closed cavity it can be shown that the total Abraham light momentum (${\int} {{\mathrm {d}}V} \,{\mathbf{S}}/c^2$) of a mode always vanishes, and thereby the angular momentum ${\cal L}$ is origin independent.

In the Supplementary Note 1, it is shown that for a sufficiently large cavity (*d* ≪ *L*_*i*_, *i* = 1, 2) the *z*-component of the angular momentum is

1 $$\frac{{{\it{{\cal L}}}_z}}{{A_{{\mathrm{tot}}}}} \approx s\frac{2}{{c^2l_{\mathrm{P}}}}\left| {\mathop {\int}\limits_{{\mathrm{cav}}{\mathrm{.}}\,{\mathrm{perimeter}}} {{\mathrm {d}}x_{||}{\int} {{\mathrm {d}}x_ \bot } {\int} {{\mathrm {d}}z} \,S_{||}} } \right|,$$

where *A*_tot_ = *L*_1_ × *L*_2_ is the cross-sectional area, *l*_p_ = 2(*L*_1_ + *L*_2_) is the cavity perimeter, and *s* = +1 (*s* = −1) for a mode that circulates in the anti-clockwise (clockwise) direction, respectively. The coordinate $x_{||}$ is measured along the cavity perimeter and the coordinate $x_ \bot$ along the perpendicular direction ($x_{||}$ and $x_ \bot$ are in the *xoy* plane). Furthermore, $S_{||}$ is the Poynting vector component parallel to the cavity walls. Similar to the theory of photonic crystals^33^, the spatially averaged Poynting vector along the propagation path can be related to the (net) group velocity *v*_g_ of the edge-wave and with the energy stored in the cavity ${\cal E}$ as follows: $\mathop {\int}\limits_{{\mathrm{cav}}{\mathrm{.}}\,{\mathrm{perimeter}}} {{\mathrm {d}}x_{||}} {\int} {{\mathrm {d}}x_ \bot } {\int} {{\mathrm {d}}z} \,S_{||} = v_{\mathrm{g}}{\it{{\cal E}}}$ (see the Supplementary Note 2 and the Supplementary Figure 1). Hence, the angular momentum can be written as

2 $$\frac{{{\it{{\cal L}}}_z}}{{A_{{\mathrm{tot}}}}} \approx s\frac{2}{{c^2l_{\mathrm{P}}}}\left| {v_{\mathrm{g}}} \right|\cal{E}.$$

The correction term that makes the two sides of the equation identical vanishes in the limit *A*_tot_ → ∞. In practice, the equation holds true when the cavity diameter is a few times larger than the characteristic modal size of the edge wave.

### Angular momentum of thermal light

Suppose now that the system is in thermodynamic equilibrium with a reservoir at temperature *T*, and that all the light inside the cavity is generated by thermal (or quantum) fluctuations. Crucially, a few recent works^18,32,34,35^ have shown that a thermodynamic equilibrium (without any heat sources) is compatible with a circulation of thermal energy in closed orbits. Specifically, for nonreciprocal systems the expectation of the heat current^18,35^ and of the angular momentum^18^ can be nontrivial. In the limit of vanishingly small material loss, the angular momentum expectation can be written as $\left\langle {{\cal L}_z} \right\rangle = \mathop{\sum}\limits_{{\omega} _n > 0} {\cal{E}}_{T,{\omega}_n}{\cal{L}}^{(n)}$ where the summation is over all the positive frequency modes of the cavity. Here, ${\cal L}^{(n)}$ is the angular momentum of a generic mode normalized to its energy and *ω*_*n*_ is the oscillation frequency. The formula of $\left\langle {{\cal L}_z} \right\rangle$ is consistent with the fluctuation-dissipation theorem (see ref. ^18^). The (unilateral) angular momentum spectral density ${\cal L}_\omega$ is defined such that $\left\langle {{\cal L}_z} \right\rangle = \mathop {\int}\limits_0^\infty {d\omega } \,{\cal L}_\omega$. It has the explicit expression ${\cal L}_\omega = {\cal{E}}_{T,\omega }\mathop {\sum}\limits_{\omega _n > 0} {{\cal L}^{(n)}\delta (\omega - \omega _n)}$. The angular momentum expectation may be nontrivial only when the time-reversal symmetry is broken, because otherwise the heat current vanishes ($\left\langle {{\mathbf{S}}/c^2} \right\rangle = 0$) in all points of the cavity^18,35^.

In a band-gap all the cavity modes must be edge-waves. Using Eq. (2) to evaluate the angular momentum (${\cal L}^{(n)}$) of a generic edge-state it is found that:

3 $$\frac{{{\it{{\cal L}}}_\omega }}{{A_{{\mathrm{tot}}}}} \approx {\cal{E}}_{T,\omega }\frac{2}{{c^2l_{\mathrm{P}}}}\mathop {\sum}\limits_n {s_n\left| {v_{{\mathrm{g}},n}} \right|\delta (\omega - \omega _n)} ,\quad \quad ({\mathrm{in}}\,{\mathrm{a}}\,{\mathrm{band}} {-} {\mathrm{gap}}).$$

The summation is over all the (positive-frequency) edge modes in the spectral range of interest. Here, *v*_g*.n*_ is the group velocity of the *n*th mode and *s*_*n*_ = ±1 depending if the energy flows in the anti-clockwise or clockwise direction. As discussed in the Supplementary Note 2, the edge modes can be organized in branches (*m* = 1, 2, …), and the dispersion of each branch is of the form $\omega _m\left( {k_{||}} \right)$. The parameter $k_{||}$ determines the total phase delay ($k_{||}l_{\mathrm{P}}$) acquired by the wave as it travels one complete loop around the cavity. Even though $k_{||}$ may be regarded as a continuous variable, only solutions with $k_{||}l_{\mathrm{P}} = 2\pi n$ with *n* integer are physical. Thus, for a large cavity perimeter and for a given branch the sum over the edge-waves can be approximated by an integral: $\frac{1}{{l_{\mathrm{P}}}}\mathop {\sum}\limits_n { \to \frac{1}{{2\pi }}{\int} {{\mathrm {d}}k_{||}} }$. This shows that $\frac{{{\cal L}_\omega }}{{A_{{\mathrm{tot}}}}} \approx {\cal{E}}_{T,\omega }\frac{1}{{c^2\pi }}\mathop {\sum}\limits_m {{\int} {{\mathrm {d}}k_{||}} \,s_m\left| {v_{{\mathrm{g}},m}} \right|\delta (\omega - \omega _m)}$. But since $v_{{\mathrm{g}},m} = \frac{{\partial \omega _m}}{{\partial k_{||}}}$ (see the Supplementary Note 2), we finally conclude that:

4 $$\frac{{{\cal L}_\omega }}{{A_{{\mathrm{tot}}}}} \approx - {\cal{E}}_{T,\omega }\frac{1}{{c^2\pi }}{\it{{\cal C}}}_\omega ,\quad {\mathrm{with}}\quad {\it{{\cal C}}}_\omega = - \mathop {\sum}\limits_{\omega _m = \omega } {s_m} .$$

The sum is over all the edge-modes for which *ω*_*m*_ = *ω* (each branch may contribute with one or more points). Since *s*_*m*_ = ± 1 the parameter ${\cal{C}}_\omega$ simply counts the difference between the number of edge modes associated with an anti-clockwise power flow (*s*_*m*_ = +1) and the number of edge modes associated with a clockwise power flow (*s*_*m*_ = −1) at frequency *ω*. In particular, we see that the absolute value of the sum gives the net number of unidirectional modes. This is the first key result of the article. It establishes that in the band-gaps of a photonic insulator cavity, the expectation of the (Abraham) angular momentum spectral density per unit of area is quantized in units of $\frac{1}{{\pi c^2}}{\cal{E}}_{T,\omega }$. The quantization is strictly valid in the continuum limit, *A*_tot_ → ∞ (likewise, in electronic systems the Hall conductivity is quantized only when the sample area approaches infinity). The quantized angular momentum density is determined by the net number of unidirectional edge modes (${\cal C}_\omega = 0, \pm 1, \pm 2, \ldots$) at frequency *ω*. This property is rather general and does not depend on the topological nature of the system. Defects may lead to additional contributions to the angular momentum, but their presence can be safely neglected in state-of-the-art photonic designs. Furthermore, it is shown in the Supplementary Note 3 that the topological cavity may be regarded as a circular transmission line and that the power spectral density associated with the energy flow around the cavity walls is also quantized.

### The angular momentum quantum

Let us now focus on topological Chern-type materials^14,15,36,37^. The bulk-edge correspondence principle establishes that the net number of unidirectional edge modes at the interface of two topological materials is coincident with the gap Chern number difference^6,37^. Thus, supposing that the cavity walls are topologically trivial, (e.g., PEC walls) it follows that $\left| {{\cal C}_\omega } \right| = | {{\cal C}_{{\mathrm{gap}}}} |$, where ${\cal{C}}_{\mathrm{gap}}$  is the gap Chern number of the photonic insulator. This is the second key result of the article. It implies that the photonic Chern number has a precise physical meaning: it determines the quantized angular momentum spectral density of a cavity in a thermodynamic equilibrium. In particular, we see that the fluctuation-induced angular momentum spectral density is totally insensitive to any variation of the system parameters (e.g., a change of the biasing magnetic field) that does not close the gap. Note that the Chern number can be unambiguously defined for fully 3D waveguide platforms^38,39^. Furthermore, a more sophisticated analysis reported elsewhere demonstrates that one has precisely ${\cal{C}}_\omega = {\cal{C}}_{\mathrm{gap}}$^40^ (the numerical examples presented in the following confirm this result; here, ${\cal{C}}_{\mathrm{gap}}$  is the sum of the Chern numbers of the bands below the gap, including negative frequency bands. The Chern number of a given band is defined consistently with refs. ^36,38^: ${\cal{C}}_n = {\hat{\mathbf z}} \cdot \nabla _{\mathbf{k}} \times {\cal{A}}_{n{\mathbf{k}}}$ with the Berry potential given by ${\cal{A}}_{n{\mathbf{k}}} = i\langle {\mathbf{Q}}_{n{\mathbf{k}}}|\partial _{\mathbf{k}}{\mathbf{Q}}_{n{\mathbf{k}}}\rangle$). Thus, a positive (negative) gap Chern number implies that the unidirectional edge waves propagate clockwise (anti-clockwise) with respect to the *z*-axis.

### Magnetized electric plasma cavity

To illustrate the application of the developed ideas, we take the photonic insulator as a magnetized electric plasma with a gyrotropic permittivity response, $\bar \varepsilon = \varepsilon _{\mathrm{t}}{\mathbf{1}}_{\mathrm{t}} + {\varepsilon} _{\mathrm{a}}{\hat{\mathbf z}} \otimes {\hat{\mathbf z}} + i{\varepsilon} _{\mathrm{g}}{\hat{\mathbf z}} \times 1$, with ${\mathbf{1}}_{\mathrm{t}} = {\hat{\mathbf x}} \otimes {\hat{\mathbf x}} + {\hat{\mathbf y}} \otimes {\hat{\mathbf y}}$ and

5 $$\varepsilon _{\mathrm{t}} = 1 - \frac{{\omega _{\mathrm{p}}^2(1 + i\gamma /\omega )}}{{(\omega + i\gamma )^2 - \omega _{\mathrm{c}}^2}},\,\varepsilon _{\mathrm{a}} = 1 - \frac{{\omega _{\mathrm{p}}^2}}{{\omega (\omega + i\gamma )}},\,{\mathrm{and}}\\ \varepsilon _{\mathrm{g}} = \frac{1}{\omega }\frac{{\omega _{\mathrm{c}}\omega _{\mathrm{p}}^2}}{{\omega _{\mathrm{c}}^2 - (\omega + i\gamma )^2}}$$

where *ω*_p_ is the plasma frequency, *γ* is the collision frequency, $\omega _{\mathrm{c}} = - qB_0/m$ is the cyclotron frequency (*ω*_c_ > 0 when the magnetic field is directed along +*z*), *q* = −*e* is the electron charge and *m* is the electron effective mass^41^. The bias magnetic field is ${\mathbf{B}} = B_0{\hat{\mathbf z}}$. InSb with a magnetic bias and other narrow-gap semiconductors have a similar gyrotropic response at terahertz frequencies^42,43^.

**Fig. 2:**
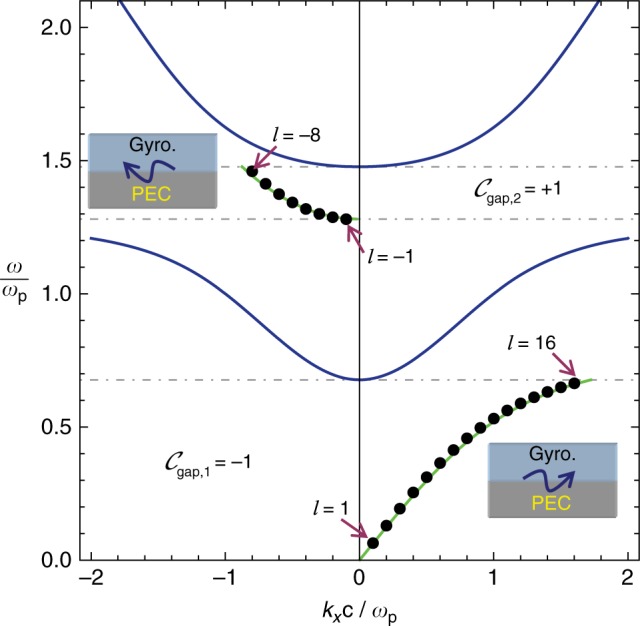
Dispersion of the bulk modes and of the edge waves. Blue lines: band diagram of the bulk gyrotropic material with $\omega _{\mathrm{c}} = 0.8\omega _{\mathrm{p}}$. The two photonic band-gaps are delimited by the dashed horizontal grid lines. The gap Chern numbers (${\cal{C}}_{\mathrm{gap,1}}$ and ${\cal{C}}_{\mathrm{gap,2}}$) are given in the insets. Green lines: dispersion of the (gap) edge modes supported by a planar interface (*y* = 0) between the gyrotropic material (region *y* > 0) and PEC material (region *y* < 0). The edge modes propagate along the *x*-axis (see the insets). The edge mode dispersion is shown only in the band-gaps. Discrete black symbols: resonant frequencies of a cylindrical cavity ($R = 10c/\omega _{\mathrm{p}}$) with PEC walls filled by the gyrotropic material. The modes are labeled by the azimuthal quantum number *l*

Figure 2 shows the band structure of a magnetized plasma with *ω*_c_ = 0.8*ω*_p_ (blue lines) for propagation in the *xoy* plane and transverse-magnetic (TM) polarization (the nontrivial field components are *E*_*x*_, *E*_*y*_, *H*_*z*_). The medium has two photonic band-gaps. It is known that lossless electromagnetic continua may be topologically classified^36^, and in particular a lossless magnetized plasma (*γ* = 0^+^) has topologically nontrivial phases^18,44,45^. The gap Chern numbers ${\cal{C}}_{{\mathrm{gap}},i}$ indicated in the insets of Fig. 2 are calculated as detailed in refs. ^36,38^. They include the contributions of all bands below the band-gap, including the negative frequency bands^38^ (not shown in Fig. 2). The dispersion of the edge-states supported by a planar interface of the magnetized plasma (region *y* > 0) and a PEC material (region *y* < 0) is represented by the green lines in Fig. 2. Consistent with the bulk-edge correspondence principle, each band-gap supports a unidirectional edge-wave.

**Fig. 3:**
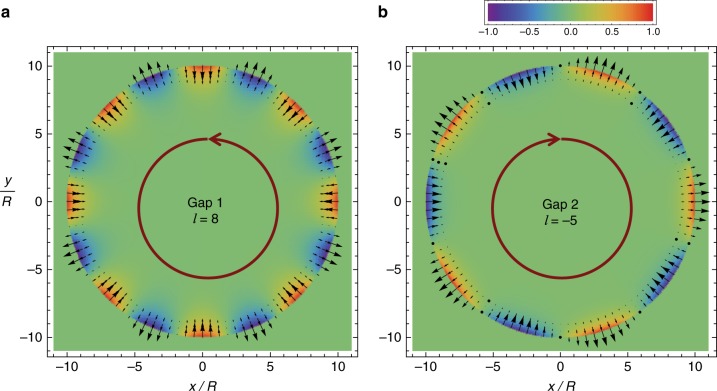
Edge modes of the topological cavity. Each panel shows a density plot of a time snapshot of the magnetic field in a cylindrical cavity ($R = 10c/\omega _{\mathrm{p}}$) for (**a**) the mode *l* = 8 in the 1st (low-frequency) gap (propagating in the anti-clockwise direction) and (**b**) the mode *l* = −5 in the 2nd (high-frequency) gap (propagating in the clockwise direction). The arrows represent the electric field lines

Consider now a cylindrical cavity filled with the magnetized plasma. The cavity lateral walls are PEC. Furthermore, to ease the analytical treatment the cavity cross-section is circular with radius *R*. In the Supplementary Note 4, it is shown that the cavity modes have a magnetic field of the form $H_z = H_0I_{\left| l \right|}(\alpha _{{\mathrm{ef}}}\rho ){\mathrm {e}}^{il\varphi }$, with $I_{\left| l \right|}$ the modified Bessel function of the 1st kind, $l = 0, \pm 1, \ldots$ is the azimuthal quantum number, $\alpha _{{\mathrm{ef}}} = \sqrt { - \varepsilon _{{\mathrm{ef}}}} \omega /c$ and $\varepsilon _{{\mathrm{ef}}} = ( {\varepsilon _{\mathrm{t}}^2 - \varepsilon _{\mathrm{g}}^2} )/\varepsilon _{\mathrm{t}}$ is the effective permittivity of the gyrotropic material. The cavity modes satisfy the dispersion equation: $\alpha _{{\mathrm{ef}}}R\frac{I{{\prime}_{\hskip -2pt \left| l \right|}\left( {\alpha _{{\mathrm{ef}}}R} \right)}}{{I_{\left| l \right|}\left( {\alpha _{{\mathrm{ef}}}R} \right)}} - l\frac{{\varepsilon _{\mathrm{g}}}}{{\varepsilon _{\mathrm{t}}}}\, = 0$. The resonant frequencies for a cavity with radius $R = 10c/\omega _{\mathrm{p}}$ are represented in Fig. 2 as discrete black dots, in the spectral range determined by the band-gaps. As seen, the discrete dots follow closely the dispersion of the edge states associated with a planar interface (green lines), indicating that in the band-gaps the cavity modes are indeed localized near the lateral walls. This property is confirmed by Fig. 3a and 3b, which represent the profiles of two generic cavity modes in the 1st (low-frequency) and 2nd (high-frequency) band-gaps, respectively. The modes of the 1st (2nd) gap have a positive (negative) azimuthal quantum number and circulate in the anti-clockwise (clockwise) direction.

**Fig. 4:**
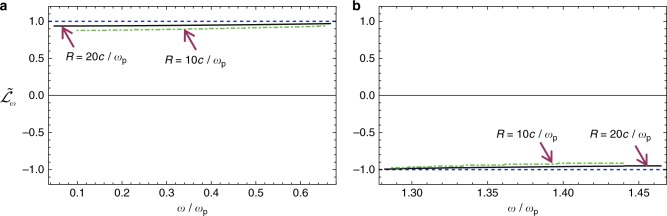
Angular momentum spectral density as a function of frequency. The normalized angular momentum spectral density $\tilde {\cal L}_\omega$ is depicted for (**a**) the 1st band gap and (**b**) for the 2nd band gap for a cylindrical cavity with radius $R = 10c/\omega _{\mathrm{p}}$ (green dot-dashed line) and $R = 20c/\omega _{\mathrm{p}}$ (black line). As *R* → ∞, the angular momentum density becomes quantized (blue dashed horizontal line). The cyclotron frequency is $\omega _{\mathrm{c}} = 0.8\omega _{\mathrm{p}}$

The angular momentum spectral density was numerically calculated using the formula ${\cal L}_\omega = {\cal{E}}_{T,\omega }\mathop {\sum}\limits_{\omega _n > 0} {{\cal L}^{\left( n \right)}\delta (\omega - \omega _n)}$. An explicit expression for the normalized angular momentum of a generic mode (${\cal L}^{\left( n \right)}$) is given in the Supplementary Note 4. The *δ*-function is spread in the interval $(\omega _n + \omega _{n - 1})/2 < \omega < (\omega _{n + 1} + \omega _n)/2$ as a rectangular pulse with height $2/(\omega _{n + 1} - \omega _{n - 1})$. Figure 4a and b depict the normalized spectral density $\tilde {\cal L}_\omega \equiv \frac{{{\cal L}_\omega c^2\pi }}{{{\cal E}_{T,\omega }A_{{\mathrm{tot}}}}}$ in the 1st and 2nd band-gaps, respectively, for cavities with radius $R = 10c/\omega _{\mathrm{p}}$ (dot-dashed green lines) and $R = 20c/\omega _{\mathrm{p}}$ (black lines). As seen, even though the cavity radius is only a few free-space wavelengths, the numerical results are consistent with the approximate identity (4): $\tilde {\cal L}_\omega \approx - {\cal C}_\omega = + 1$ in the 1st gap and $\tilde {\cal L}_\omega \approx - {\it{{\cal C}}}_\omega = - 1$ in the 2nd gap. In *R* → ∞ limit the approximate identities become exact, and the angular momentum spectral density is exactly quantized and is determined by the gap Chern numbers: $\tilde {\cal L}_\omega = - {\cal C}_{{\mathrm{gap,1}}}$ in the 1st gap and $\tilde {\cal L}_\omega = - {\cal C}_{{\mathrm{gap,2}}}$ in the 2nd gap (dashed blue lines in Fig. 4).

**Fig. 5:**
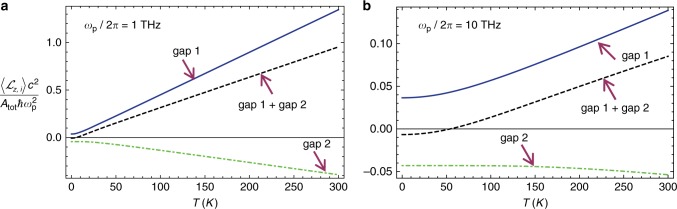
Fluctuation induced angular momentum $\langle {{\cal L}_{z,i}} \rangle$ as a function of the temperature. The cylindrical cavity has radius $R > > c/\omega _{\mathrm{p}}$ and the plasma frequency is (**a**) $\omega _{\mathrm{p}}/2\pi = 1\,{\mathrm{THz}}$ and (**b**) $\omega _{\mathrm{p}}/2\pi = 10\,{\mathrm{THz}}$. The cyclotron frequency is $\omega _{\mathrm{c}} = 0.8\omega _{\mathrm{p}}$. Solid blue line: contribution from the 1st (low-frequency) band gap. Dot-dashed green line: contribution from the 2nd (high-frequency) band gap. Dashed black line: combined contribution of the two band gaps. Note that the contribution of the modes outside the spectral region of the band-gaps is not included in the calculation

Figure 5 represents the total angular momentum contribution of each band gap, defined as $\langle {{\cal L}_{z,i}} \rangle = \mathop {\int}\limits_{{\mathrm{gap}}\,{\mathrm{i}}} {{\mathrm {d}}\omega } \,{\cal L}_\omega$, as a function of the temperature for $\omega _{\mathrm{p}}/2\pi = 1\,{\mathrm{THz}}$ and $\omega _{\mathrm{p}}/2\pi = 10\,{\mathrm{THz}}$. As could be anticipated, the system angular momentum due to the edge modes increases approximately linearly with the temperature because ${\cal{E}}_{{{T}},\omega } {\approx {k}}_{\mathrm{B}}{{T}}$ for large *T*. The contributions of the band-gaps to the angular momentum have opposite signs. For very low-temperatures the contribution of the 2nd (high-frequency) gap dominates, and the total angular momentum due to edge waves is negative ($\langle {{\cal L}_{z,1}} \rangle + \langle {{\cal L}_{z,2}}\rangle < 0$). In the *T*→0^+^ limit, the angular momentum is exclusively due to the quantum vacuum fluctuations. In this case, the zero-point energy (due to the gap edge-modes) flows in the clockwise direction around the lateral walls, i.e., in the direction determined by the electron “skipping orbits” (opposite to the bulk cyclotron orbits)^18,46,47^. In contrast, for moderately large temperatures the thermal-effects make $\langle {{\cal L}_{z,1}} \rangle + \langle {{\cal L}_{z,2}} \rangle$ positive and the contribution of the 1st (low-frequency) gap dominates. It should be noted that bulk modes may also contribute to the fluctuation-induced angular momentum^48,49^, and hence $\langle {{\cal L}_{z,1}} \rangle + \langle {{\cal L}_{z,2}} \rangle$ only gives a partial (band-gap) component of $\left\langle {{\cal L}_z} \right\rangle$.

### Outline of a microwave experiment

**Fig. 6:**
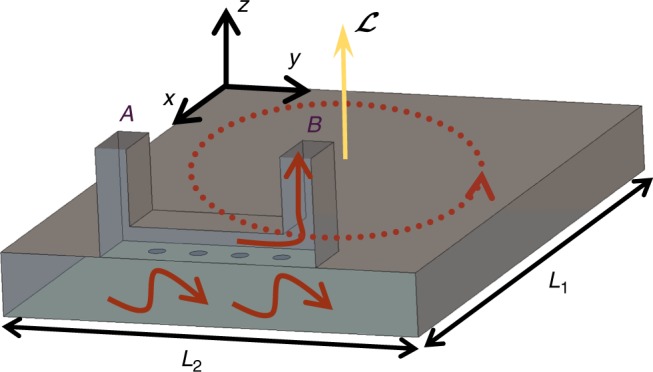
Setup of a possible experiment at microwave frequencies. A metallic cavity is filled with a topological photonic crystal (not represented in the figure; e.g., a photonic crystal formed by ferrite rods^8^) and is connected to a waveguide coupler through multiple apertures on the top metallic plate. The directional coupler can be designed in such a manner that for an anti-clockwise (clockwise) flow of thermal energy the coupled signal is transmitted mainly to port B (port A). The front wall of the cavity and the front wall of the directional coupler are not shown in order to visualize the interior of the structure

Next, we outline a possible microwave experiment to verify that the fluctuation-induced angular momentum density is nontrivial. Figure 6 depicts the metallic cavity filled with a topological material. A waveguide directional coupler is connected to the cavity through a multihole aperture (similar to a Bethe hole coupler)^50^. In a microwave experiment (e.g., $\omega /2\pi \sim 10\,{\mathrm{GHz}}$), the topological material can be implemented with a square array of ferrite rods biased with a static magnetic field, similar to ref. ^8^. A small fraction of the thermal energy flowing near the cavity walls may be transferred to either port A or port B of the directional coupler. The directional coupler can be designed in such a manner that when the flow of thermal energy follows an anti-clockwise (clockwise) motion (with respect to the *z*-axis) most of the energy is coupled to the port B (port A), and the other port is isolated^50^.

A measurement must perturb slightly the thermal equilibrium because otherwise the Poynting vector orbits are closed and the thermal flow is impossible to detect (in particular, the zero-point energy part of ${\cal{E}} _{{{T}},\omega }$ cannot be directly measured)^18^. Hence, in an experiment the ports A and B must be held at a temperature *T*_0_ different from the cavity temperature *T*. A detailed analysis reported in the Supplementary Note 5 shows that the difference between the power flows at ports A and B when the thermal equilibrium is perturbed (*T*_0_ < *T*) is given by

6 $$\delta p \equiv p_{\mathrm{B}} - p_{\mathrm{A}} \approx k_{\mathrm{B}}(T - T_0){\mathrm{\Delta }}C_{\mathrm{D}}\frac{{{\mathrm{\Delta }}\omega }}{{2\pi }},$$

where Δ*ω* is the bandwidth of the detecting circuit. The analysis assumes that both ports A and B are terminated by matched loads and that the material dissipation in the directional coupler is negligible due to the weak loss of metals at microwaves. The coefficient Δ*C*_D_ is determined by the difference of the coupling strengths between ports A and B and all the cavity modes; it may be found with an independent calibration process, specifically from the *S*-parameters of the two-port network. Equation (6) is rather general and applies even when the frequency of interest is outside a band-gap and for any cavity size.

In the following, we concentrate on the case in which the detecting circuit bandwidth lies in a band-gap, so that Δ*C*_D_ is entirely determined by the edge modes. Interestingly, because the power density transported by the edge modes is quantized and is independent of the system area (Supplementary Note 5), it follows that Δ*C*_D_ will depend very little on the cavity cross-sectional area provided its diameter is a few times larger than the characteristic modal size so that the edge waves attached to opposite walls are well spatially separated. The typical modal size of the edge modes for a topological photonic crystal is on the order of the lattice constant^8^. Furthermore, it will also depend little on the specific location of the directional coupler in the cavity. Thus, a fingerprint of the topological gap is that $\delta p$ must be nearly independent of the cavity cross-section and of the location of the directional coupler. Note that outside a band-gap these properties do not hold.

For a reciprocal cavity its coupling with the two ports is symmetric and hence Δ*C*_D_ vanishes and $\delta p = 0$. Similarly, for a topologically trivial (but not necessarily reciprocal) gap (${\cal{C}}$ = 0) the number of edge states (if nonzero) circulating in counterclockwise direction is identical to the number of states circulating in the clockwise direction. This indicates that Δ*C*_D_ is negligible when ${\cal{C}}$ = 0. Clearly, a significant imbalance between the net energy fluxes at ports A and B is only possible when ${\cal{C}}$ ≠ 0, and it will provide a clear signature of a nontrivial topology and confirm the circulation of thermal energy in closed orbits.

Ideally the directional coupler should ensure that the power transported by the modes circulating along a given direction is delivered to a single port. For example, if the coupler is designed in such a way that when the edge-modes propagate in an anti-clockwise (clockwise) direction most of the thermal energy is coupled to port B (A) then ${\mathrm{\Delta }}C_{\mathrm{D}} = - {\mathrm{sgn}}({\cal C})\left| {{\mathrm{\Delta }}C_{\mathrm{D}}} \right|$. Thus, the sign of $\delta p$ is linked to the sign of the Chern number. Furthermore, for an ideal coupler $\left| {{\mathrm{\Delta }}C_{\mathrm{D}}} \right|$ represents the fraction of thermal energy rerouted from the cavity to the coupled port. Standard couplers can be very directive such that the ports coupling strength differs by four orders of magnitude^50^.

At ports A and B the thermal power may be collected by a microwave bolometer (radiometer)^51,52^. It is shown in the Supplementary Note 5, that the power rerouted from the topological cavity to the coupled port increases the noise temperature of the corresponding bolometer by $\delta T_{\mathrm{n}} \approx T\left| {{\mathrm{\Delta }}C_{\mathrm{D}}} \right|$, where *T* is the temperature of the cavity. For $\left| {{\mathrm{\Delta }}C_{\mathrm{D}}} \right|\sim 0.1$ and $T = 300\,{\mathrm {K}}$ the excess of noise temperature on the detector is $\delta T_{\mathrm{n}} \approx 30\,{\mathrm {K}}$. Thus, the bolometer needs to sufficiently sensitive to detect variations of the noise temperature on the order of some tens of Kelvin. Detectors cooled to a temperature on the order of *T*_0_ = 4 K can detect an excess of noise temperature as small as ~1 K and are of widespread use in radio-astronomy^53,54^. Therefore, the proposed experiment seems to be within reach using cooled state-of-art bolometers with $T_0 \le \delta T_{\mathrm{n}}$.

## Discussion

In summary, it was demonstrated that the fluctuation-induced angular momentum in a generic photonic insulator cavity with opaque-type boundaries has a quantized angular momentum in the photonic band-gaps. The quantized spectral-density depends on the net number of unidirectional edge-states at the cavity walls. For topological systems, the angular momentum is determined by the photonic Chern number, which thereby has a precise physical meaning as a “quantum” of the light-angular momentum spectral density. For ${\Bbb Z}_2$ topological photonic insulators with the time-reversal symmetry^9^ the spin-filtered ${\cal L}_\omega /A_{{\mathrm{tot}}}$ is also quantized, but the contributions of the different “spins” cancel out. The nontrivial fluctuation-induced angular momentum may be experimentally verified by coupling the light that circulates around the cavity walls to a directional coupler and by detecting the imbalance between the energy sensed by the different arms of the coupler. We believe that the interpretation of the Chern number as a quantum of the angular momentum spectral density provides a deeper understanding about the intriguing role of topology in photonic systems^40^.

## Supplementary information

Supplementary Information

## Data Availability

The numerical data supporting the results of this article are available from the author upon request.
