# Supplementary material for: Quantized angular momentum in topological optical systems
Source: Nat Commun. 2019 Jan 21;10:349. doi: 10.1038/s41467-018-08215-5 (PMC6341178; doi:10.1038/s41467-018-08215-5)
Supplement: Supplementary file 1 — Supplementary Information [file 41467_2018_8215_MOESM1_ESM.pdf]

Supplementary Information

Quantized Angular Momentum in Topological Optical Systems

Silveirinha et al.

Supplementary Figures:

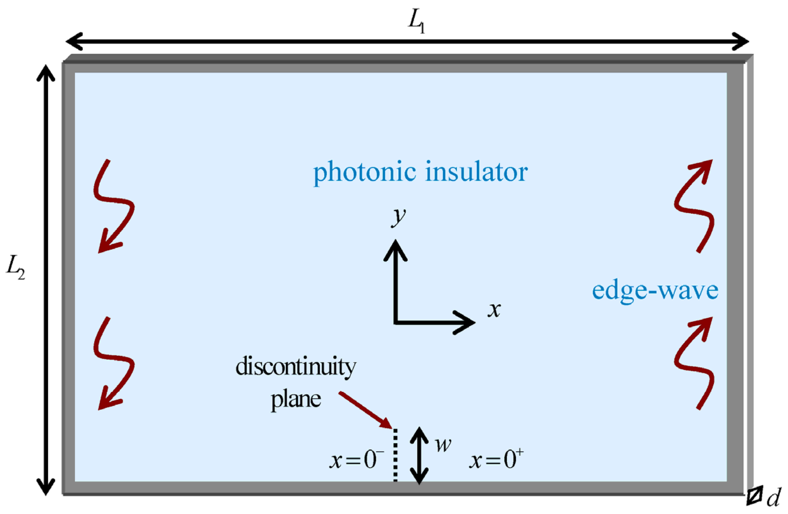

Fig. 1 **Geometry of the problem formulated in Supplementary Note 2**. A discontinuity plane (represented by the dashed line) is inserted near one of the lateral walls. Bloch-type boundary conditions are enforced at the discontinuity plane [Supplementary Eq. (6)]. The edge modes propagate around the cavity perimeter and cannot penetrate into the bulk region, which is a photonic insulator.

Supplementary Notes:

Supplementary Note 1: Derivation of the angular momentum of an edge-mode

Let us consider a closed lossless cavity with an edge-mode circulating around the lateral walls (Fig. 1 of the main text). The edge mode is described by the complex-valued electromagnetic field $(\mathbf{E},\mathbf{H})$ (the time-variation $e^{-i\omega t}$ is implicit). The Abraham angular momentum is by definition:

$$\mathcal{L}_z = \frac{1}{c^2}\hat{\mathbf{z}}\cdot\int dV\,\mathbf{r}\times\mathbf{S}, \qquad (1)$$

where $\mathbf{S}=\mathrm{Re}\left\{\mathbf{E}\times\mathbf{H}^*\right\}$ is the Poynting vector of a complex-valued field (the time-averaged Poynting vector of the real-valued field $\mathrm{Re}\left\{\mathbf{E}e^{-i\omega t}\right\}$ differs by a $1/2$ factor from $\mathrm{Re}\left\{\mathbf{E}\times\mathbf{H}^*\right\}$).

In the general case, the bulk region is a photonic crystal and thus is formed by many identical cells (let us say with dimensions $a_1\times a_2\times a_3$). The Poynting vector may vary considerably on the scale of each unit cell. Since the position vector $\mathbf{r}$ varies slowly on this scale, for a large cavity we may write:

$$\mathcal{L}_z \approx \frac{1}{c^2}\hat{\mathbf{z}}\cdot\int dV\,\mathbf{r}\times\mathbf{S}_{\mathrm{av}}, \qquad (2)$$

where $\mathbf{S}_{\mathrm{av}}$ should be understood as a spatially averaged Poynting vector with the fluctuations on the scale of the unit cell removed.

We arbitrarily choose the origin of the coordinate axes to be at the center of the cavity. For a large cavity, the edge mode fields are concentrated near the walls and the coupling between different walls is negligible (the contribution of the corners region to

the angular momentum is negligible). Hence, $\mathcal{L}_z$ may be regarded as a sum of 4 parcels, with each parcel associated with a specific wall. Let us focus on the wall $x = L_1/2$, which gives the angular momentum contribution $\mathcal{L}_z\big|_{\substack{\text{wall}\\ x=L_1/2}} = \frac{1}{c^2}\hat{\mathbf{z}} \cdot \int_{\tilde{V}} dV \; \mathbf{r} \times \mathbf{S}_{\text{av}}$, where $\tilde{V}$ is some volumetric region nearby the considered wall of the form $L_1/2 - \delta_{\text{s}} \le x \le L_1/2$ and $-L_2/2 \le y \le L_2/2$. Here, δ_{s} is some characteristic penetration depth of the edge mode into the bulk region (the mode has an exponentially decay in the direction perpendicular to the wall). It is implicit that $\delta_{\text{s}} << L_i$ (i=1,2) so that $\tilde{V}$ does not overlap the edge mode profile associated with other walls. Clearly, $\mathbf{S}_{\text{av}}$ must be predominantly oriented along the $\pm\hat{\mathbf{y}}$ direction, because the energy can only flow along directions parallel to the wall. Thus, to leading order it is possible to write

$$\mathcal{L}_z\big|_{\substack{\text{wall}\\ x=L_1/2}} \approx \frac{1}{c^2}\frac{L_1}{2}\int_{\tilde{V}} dV\, S_{\text{av},y} = \frac{1}{c^2}\frac{L_1}{2}\int_{\tilde{V}} dV S_y \,. \tag{3}$$

The second identity follows from the fact that the volume integral of a spatially averaged quantity is simply the volume integral of that quantity. One can further write, $\int_{\tilde{V}} dV S_y = \int_{-L_2/2}^{L_2/2} dy \left(\int_{L_1/2-\delta_{\text{s}}}^{L_1/2} dx \int dz\, S_y \right)$. The inner integral gives the flux of the Poynting vector through each section $y = const.$, and thereby from the conservation of energy must be independent of y. This shows that:

$$\mathcal{L}_z\big|_{\substack{\text{wall}\\ x=L_1/2}} \approx \frac{1}{c^2}\frac{L_1 L_2}{2} \int_{L_1/2-\delta_{\text{s}}}^{L_1/2} dx \int dz\, S_y \,. \tag{4}$$

Proceeding in the same way for the other 3 walls, we obtain similar formulas. Generically, the contribution of a given wall is of the form $\frac{1}{c^2}\frac{A_{\text{tot}}}{2}\int dx_\perp \int dz\, S_{||}$, where $x_\perp$ is the coordinate perpendicular to the interface and $S_{||}$ is the Poynting vector component parallel to the interface (positive when the Poynting vector is oriented in the anti-clockwise direction, with respect to *z*). For a large cavity, the integral $\int dx_\perp \int dz\, S_{||}$ is independent of the considered section-cut (around the cavity perimeter) due to the conservation of energy. Hence,

$$\begin{aligned}\frac{\mathcal{L}_z}{A_{\text{tot}}} &\approx \frac{2}{c^2}\int dx_\perp \int dz\, S_{||} \\ &= s\frac{2}{c^2 l_{\text{P}}}\left|\int_{\text{cav. perimeter}} dx_{||}\int dx_\perp \int dz\, S_{||}\right|,\end{aligned} \tag{5}$$

where $s = \pm 1$ for modes that circulate in the anti-clockwise (clockwise) direction and $l_{\text{P}} = 2\left(L_1 + L_2\right)$ is the cavity perimeter.

Supplementary Note 2: Edge-mode branches and group velocity

In order to characterize the edge modes supported by a closed cavity, we introduce an auxiliary mathematical problem whose geometry is coincident with the original one, except that the fields are allowed to be discontinuous near some rectangular cut ($x = 0, -L_2/2 < y < -L_2/2 + w$, and $0 < z < d$) near the inferior lateral wall (see the Supplementary Figure 1). We will refer to this region simply as the "discontinuity plane". It is implicit that the oscillation frequency is in a band-gap of the bulk region. The width *w* should be large enough so that it exceeds the penetration depth of the edge modes into

the bulk region ($w > \delta_s$) and at the same time $w << L_2 / 2$ (thus, the cavity needs to be sufficiently large).

We are interested in the eigen-solutions of the Maxwell's equations such that the fields at the "+" and "–" sides of the discontinuity plane are linked by Bloch-type boundary conditions:

$$\mathbf{E}_{\tan}\big|_{x=0^-} = \mathbf{E}_{\tan}\big|_{x=0^+} e^{ik_{\|}l_{\mathrm{P}}}, \qquad \mathbf{H}_{\tan}\big|_{x=0^-} = \mathbf{H}_{\tan}\big|_{x=0^+} e^{ik_{\|}l_{\mathrm{P}}}. \qquad (6)$$

The subscript "tan" refers to the field components tangential to the discontinuity plane. The parameter $k_{\|}$ determines the phase delay ($k_{\|}l_{\mathrm{P}}$) acquired by the wave as it goes around the lateral walls of the cavity with perimeter $l_{\mathrm{P}} = 2(L_1 + L_2)$. Evidently, solutions with $k_{\|}l_{\mathrm{P}} = 2\pi n$ and n integer are also solutions of the original problem with no discontinuity plane. However, here it is convenient to admit that $k_{\|}$ can take any real value. Furthermore, by analytic continuation, we can also consider eigen-solutions with $k_{\|}$ complex, $k_{\|} = k' + ik''$, which are evidently associated with some complex-valued eigen-frequencies $\omega = \omega' + i\omega''$.

Let us then consider a generic family of natural modes $\left(\mathbf{E}_{k_{\|}}, \mathbf{H}_{k_{\|}}\right)$ of the Maxwell's equations in the cavity that satisfy the Bloch-type boundary conditions (6) with dispersion $\omega = \omega(k_{\|})$.

For a lossless cavity, the conservation of energy implies that $\nabla \cdot \mathbf{S} + \partial_t W = 0$, with $\mathbf{S}$ the Poynting vector and W the instantaneous stored energy density. For a dispersive lossless medium and complex-valued fields the Poynting vector is defined as $\mathbf{S} = \mathrm{Re}\left\{\mathbf{E} \times \mathbf{H}^*\right\}$, whereas the electromagnetic energy density may be written as

$W = \frac{1}{2}\mathbf{Q}^* \cdot \mathbf{M}_{\mathrm{g}} \cdot \mathbf{Q}$, with $\mathbf{Q}$ the state-vector of the system and $\mathbf{M}_{\mathrm{g}}$ a generalized material matrix [1, 2]. Integrating the formula $\nabla \cdot \mathbf{S} + \partial_t W = 0$ over the cavity volume, it is found that:

$$\frac{d}{dt}\int dV\, W(\mathbf{r},t) = \hat{\mathbf{x}} \cdot \int_{\substack{\text{discont.}\\ \text{plane}}} dS \left[\mathbf{S}(\mathbf{r},t)\big|_{x=0^+} - \mathbf{S}(\mathbf{r},t)\big|_{x=0^-} \right]. \tag{7}$$

For complex-valued fields with a time variation $e^{-i\omega t} = e^{-i\omega' t} e^{\omega'' t}$ both $\mathbf{S}$ and W vary in time as $e^{2\omega'' t}$. Furthermore, for modes that satisfy the supplementary Eq. (6) with a complex-valued $k_{\|} = k' + ik''$ it is evident that $\mathbf{S}(\mathbf{r},t)\big|_{x=0^-} = e^{-2k'' l_P}\mathbf{S}(\mathbf{r},t)\big|_{x=0^+}$. Hence, for a generic eigenmode it is possible to write:

$$2\omega'' \int dV\, W_{k'+ik''}(\mathbf{r},t) = \left(1 - e^{-2k'' l_P}\right)\hat{\mathbf{x}} \cdot \int_{\substack{\text{discont.}\\ \text{plane}}} dS\, \mathbf{S}_{k'+ik''}(\mathbf{r},t)\big|_{x=0^+}, \tag{8}$$

with $\omega' + i\omega'' = \omega(k_{\|})\big|_{k_{\|}=k'+ik''}$. Next, we take the limit $k'' \to 0$. Since the eigenmodes satisfy the dispersion equation $\omega = \omega(k_{\|})$, by doing a Taylor expansion it is seen that $\omega'' = \frac{\partial \omega}{\partial k_{\|}}(k')k''$. Hence, modes with a real-valued $k_{\|}$ satisfy:

$$v_{\mathrm{g}} \int dV\, W_{k'}(\mathbf{r}) = l_{\mathrm{P}}\, \hat{\mathbf{x}} \cdot \int_{\substack{\text{discont.}\\ \text{plane}}} dS\, \mathbf{S}_{k'}(\mathbf{r})\big|_{x=0^+}. \tag{9}$$

where $v_{\mathrm{g}} = \frac{\partial \omega}{\partial k_{\|}}(k')$ may be understood as the (net) group-velocity of the edge-mode. Note that for a real-valued oscillation frequency both $\mathbf{S}$ and W are independent of time. Moreover, the conservation of energy implies that the integral

$\hat{\mathbf{x}}\cdot \int\limits_{\substack{\text{discont.}\\ \text{plane}}} dS\, \mathbf{S}_{k'}\Big|_{x=0^+} = \int\limits_{\substack{\text{discont.}\\ \text{plane}}} dx_\perp dz\, S_{\|}\Big|_{x=0^+}$ is independent of the section-cut around perimeter of the cavity (see Supplementary Note 1). Thus, a generic edge-mode of the original cavity satisfies (dropping all the irrelevant subscripts):

$$\int\limits_{\text{cav. perimeter}} dx_{\|} \int dx_\perp \int dz\, S_{\|} = v_{\mathrm{g}} \int\limits_{\text{cavity}} dV\, W = v_{\mathrm{g}} \mathcal{E}\,, \tag{10}$$

where $\mathcal{E}$ is the stored energy. The notations || and $\perp$ are used with the same meaning as in Supplementary Note 1. The above formula generalizes the result $\int dV\, \mathbf{S} = \mathbf{v}_{\mathrm{g}} \int dV\, W$, satisfied by generic Bloch waves in photonic crystals [3, 4].

Supplementary Note 3: The power spectral density circulating around the cavity walls

Let p_ω be the unilateral power spectral density associated with the energy transported around the cavity walls ($P = \int_0^\infty d\omega\, p_\omega$). From the supplementary Eq. (10), the power transported by a given edge mode is:

$$P = \frac{1}{l_{\mathrm{P}}} v_{\mathrm{g}} \mathcal{E}\,, \tag{11}$$

where l_{P} is the cavity perimeter. Thus, the power spectral density in a band-gap is given by

$$p_\omega = \mathcal{E}_{T,\omega} \frac{1}{l_{\mathrm{P}}} \sum_n v_{\mathrm{g},n} \delta(\omega - \omega_n)\,, \tag{12}$$

with the summation over all the edge modes. For a sufficiently large cavity we can use $\frac{1}{l_{\mathrm{P}}} \sum_n \to \frac{1}{2\pi} \int dk_{\|}$ (see the main text). Taking into account the contributions of all edge-

mode branches and the link between the gap Chern number and the net number of unidirectional edge modes one obtains:

$$p_\omega \approx -\mathcal{C}\frac{1}{2\pi}\mathcal{E}_{T,\omega}. \tag{13}$$

Thus, it follows that p_ω is also quantized in units of $\frac{1}{2\pi}\mathcal{E}_{T,\omega}$. Note that the sign of p_ω determines the direction (anti-clockwise vs. clockwise) along which the thermal energy flows.

In simple terms, in the band gaps the cavity is analogous to a one-dimensional circular transmission line. Indeed, the fluctuation-induced power density transported by a standard transmission line mode is precisely $p_\omega^{\text{per mode}} = \pm\frac{1}{2\pi}\mathcal{E}_{T,\omega}$ [7]. In a nonreciprocal line, the net number of unidirectional modes can be nonzero, leading to a nontrivial p_ω. Note that from the definition of angular momentum it follows immediately that for a circular transmission line $\mathcal{L}_\omega = \frac{2A_{\text{tot}}}{c^2}p_\omega$.

Supplementary Note 4: Modes of a cylindrical cavity filled with a gyrotropic material

We consider a cylindrical cavity with radius *R* filled with a gyrotropic material with (relative) permittivity tensor $\bar{\varepsilon} = \varepsilon_{\text{t}}\mathbf{1}_{\text{t}} + \varepsilon_{\text{a}}\hat{\mathbf{z}}\otimes\hat{\mathbf{z}} + i\varepsilon_{\text{g}}\hat{\mathbf{z}}\times\mathbf{1}$, with $\mathbf{1}_{\text{t}} = \hat{\mathbf{x}}\otimes\hat{\mathbf{x}} + \hat{\mathbf{y}}\otimes\hat{\mathbf{y}}$, and a trivial permeability. The cavity lateral wall is a perfect electric conducting (PEC) surface. The fields are assumed to be TM-polarized with $\mathbf{H} = H_z\hat{\mathbf{z}}$ and $\mathbf{E} = E_x\hat{\mathbf{x}} + E_y\hat{\mathbf{y}}$. It is implicit that the fields are independent of the *z*-coordinate ($0 < z < d$) so that the problem is effectively two-dimensional. In the bulk region (the interior of the cavity) the magnetic field satisfies [5]:

$$\left(\nabla^2+\frac{\omega^2}{c^2}\varepsilon_{\text{ef}}\right)H_z=0\,, \qquad \text{with } \varepsilon_{\text{ef}}=\frac{\varepsilon_{\text{t}}^2-\varepsilon_{\text{g}}^2}{\varepsilon_{\text{t}}}. \tag{14}$$

Hence, adopting a system of cylindrical coordinates (ρ,φ) it is clear that a generic natural mode has a magnetic field of the form (apart from an arbitrary normalization factor):

$$\eta_0 H_z = I_{|l|}\left(\alpha_{\text{ef}}\rho\right)e^{il\varphi}\,, \tag{15}$$

where η_0 is the free-space impedance, $I_{|l|}$ the modified Bessel function of the 1st kind, $l=0,\pm1,...$ is the azimuthal quantum number and $\alpha_{\text{ef}}=\sqrt{-\varepsilon_{\text{ef}}}\,\omega/c$. The electric field can be found using $-i\omega\varepsilon_0\mathbf{E}=\overline{\varepsilon}^{-1}\cdot\nabla\times\mathbf{H}$ with $\overline{\varepsilon}^{-1}=\frac{1}{\varepsilon_{\text{ef}}}\left(\mathbf{1}_t-i\frac{\varepsilon_{\text{g}}}{\varepsilon_{\text{t}}}\hat{\mathbf{z}}\times\mathbf{1}\right)+\frac{1}{\varepsilon_{\text{a}}}\hat{\mathbf{z}}\otimes\hat{\mathbf{z}}$. Since $\nabla\times\mathbf{H}=\frac{1}{\rho}\partial_\varphi H_z\hat{\boldsymbol{\rho}}-\partial_\rho H_z\hat{\boldsymbol{\varphi}}$ it follows that:

$$\mathbf{E}=\frac{1}{i\omega\varepsilon_0\varepsilon_{\text{ef}}}\left(-\frac{1}{\rho}\partial_\varphi H_z+\frac{i\varepsilon_{\text{g}}}{\varepsilon_{\text{t}}}\partial_\rho H_z\right)\hat{\boldsymbol{\rho}}+\frac{1}{i\omega\varepsilon_0\varepsilon_{\text{ef}}}\left(\partial_\rho H_z+\frac{i\varepsilon_{\text{g}}}{\varepsilon_{\text{t}}}\frac{1}{\rho}\partial_\varphi H_z\right)\hat{\boldsymbol{\varphi}}\,. \tag{16}$$

Using the supplementary Eq. (15), we obtain the explicit formula:

$$\begin{aligned}\mathbf{E}=&\frac{1}{i\varepsilon_{\text{ef}}\omega/c}\left(-il\frac{1}{\rho}I_{|l|}\left(\alpha_{\text{ef}}\rho\right)+\frac{i\varepsilon_{\text{g}}}{\varepsilon_{\text{t}}}\alpha_{\text{ef}}I'_{|l|}\left(\alpha_{\text{ef}}\rho\right)\right)\hat{\boldsymbol{\rho}}\\&+\frac{1}{i\varepsilon_{\text{ef}}\omega/c}\left(\alpha_{\text{ef}}I'_{|l|}\left(\alpha_{\text{ef}}\rho\right)-l\frac{\varepsilon_{\text{g}}}{\varepsilon_{\text{t}}}\frac{1}{\rho}I_{|l|}\left(\alpha_{\text{ef}}\rho\right)\right)\hat{\boldsymbol{\varphi}}\end{aligned}\,. \tag{17}$$

Imposing the PEC boundary condition ($E_\varphi=0$) at $\rho=R$, one finds the dispersion equation for the natural modes:

$$\alpha_{\text{ef}}R\frac{I'_{|l|}\left(\alpha_{\text{ef}}R\right)}{I_{|l|}\left(\alpha_{\text{ef}}R\right)}-l\frac{\varepsilon_{\text{g}}}{\varepsilon_{\text{t}}}=0\,. \tag{18}$$

The Poynting vector ($\mathbf{S}=\mathrm{Re}\{\mathbf{E}\times\mathbf{H}^*\}$) of a (complex-valued) mode has the azimuthal component $S_\varphi=-\mathrm{Re}\{E_\rho H_z^*\}$. The angular momentum of the mode is $\mathcal{L}_z=\frac{1}{c^2}\int dV\,\rho S_\varphi$, which from the supplementary equations (15) and (17) can be written explicitly as (d is the height of the cavity along z):

$$\frac{\mathcal{L}_z}{d}=\frac{2\pi}{\eta_0}\frac{1}{c^2}\frac{1}{\varepsilon_{\mathrm{ef}}\omega/c}\int_0^R d\rho\,\rho\,\mathrm{Re}\left\{I_{|l|}^*\left(\alpha_{\mathrm{ef}}\rho\right)\left(l\,I_{|l|}\left(\alpha_{\mathrm{ef}}\rho\right)-\frac{\varepsilon_g}{\varepsilon_t}\alpha_{\mathrm{ef}}\rho I'_{|l|}\left(\alpha_{\mathrm{ef}}\rho\right)\right)\right\}. \quad (19)$$

The energy density of the complex–valued field is (note that for time-averaged real-valued fields one needs to multiply the right-hand side by an additional $1/2$ factor) [1, 6]:

$$W=\frac{1}{2}\varepsilon_0\left(\mathbf{E}^*\cdot\partial_\omega\left(\omega\bar{\varepsilon}\right)\cdot\mathbf{E}+\left|\eta_0 H_z\right|^2\right). \quad (20)$$

Thus, after some straightforward calculations the stored energy, $\mathcal{E}=\int dV\,W$, can be written as:

$$\frac{\mathcal{E}}{d}=2\pi\int_0^R d\rho\frac{1}{2}\varepsilon_0\rho\left(\left(\partial_\omega\left(\omega\varepsilon_{\mathrm{t}}\right)\left(\left|E_\rho\right|^2+\left|E_\varphi\right|^2\right)+2\,\partial_\omega\left(\omega\varepsilon_{\mathrm{g}}\right)\mathrm{Re}\left\{iE_\rho E_\varphi^*\right\}\right)+\left|\eta_0 H_z\right|^2\right), \quad (21)$$

where $\partial_\omega=\partial/\partial\omega$. Combining the supplementary equations (19) and (21) one finds that the angular momentum of a cavity mode normalized to its energy is given by:

$$\frac{\mathcal{L}_z}{\mathcal{E}}=\frac{1}{\omega}\frac{\frac{1}{\varepsilon_{\mathrm{ef}}}\int_0^R d\rho\,\rho\,\mathrm{Re}\left\{I_{|l|}^*\left(\alpha_{\mathrm{ef}}\rho\right)\left(l\,I_{|l|}\left(\alpha_{\mathrm{ef}}\rho\right)-\frac{\varepsilon_{\mathrm{g}}}{\varepsilon_{\mathrm{t}}}\alpha_{\mathrm{ef}}\rho I'_{|l|}\left(\alpha_{\mathrm{ef}}\rho\right)\right)\right\}}{\int_0^R d\rho\frac{1}{2}\rho\left(\left(\partial_\omega\left(\omega\varepsilon_{\mathrm{t}}\right)\left(\left|E_\rho\right|^2+\left|E_\varphi\right|^2\right)+2\,\partial_\omega\left(\omega\varepsilon_{\mathrm{g}}\right)\mathrm{Re}\left\{iE_\rho E_\varphi^*\right\}\right)+\left|\eta_0 H_z\right|^2\right)}, \quad (22)$$

with E_ρ, E_φ, H_z defined as in the supplementary equations (15) and (17). The right-hand side of the above equation determines the parameter $\mathcal{L}^{(n)}$ used in the main text.

Supplementary Note 5: Power rerouted from the cavity to the directional coupler

In the following, we characterize the net power rerouted from the topological cavity (at temperature *T*) to the arms of the directional coupler (Fig. 6 of the main text). It is supposed that the material loss in the directional coupler is negligible and that ports A and B are terminated with matched loads (microwave bolometers) cooled to a temperature $T_0 < T$. For simplicity, in the following we refer to ports A and B as ports 1 and 2, respectively.

The thermal energy radiated by port 1 ($p_{\text{rad},1}$) is $p_{\text{rad},1} = \left(1-|s_{11}|^2\right)\mathcal{E}_{T_0,\omega}^{\text{ther}}\frac{\Delta\omega}{2\pi}$, where $\Delta\omega$ is the relevant bandwidth. Here, s_{ij} are the scattering parameters of the two-port microwave network. Furthermore, $\mathcal{E}_{T,\omega}^{\text{ther}} = \frac{\hbar\omega}{2}\left[\coth\left(\frac{\hbar\omega}{2k_{\text{B}}T}\right)-1\right] = \frac{\hbar\omega}{e^{\hbar\omega/k_{\text{B}}T}-1} \approx k_{\text{B}}T$ is the mean thermal energy of a harmonic oscillator at temperature *T*. Note that $\mathcal{E}_{T,\omega}^{\text{ther}}\frac{\Delta\omega}{2\pi}$ is the thermal noise power delivered by a circuit with temperature *T* to a matched load [8].

On the other hand, the thermal energy captured by port 1 ($p_{\text{abs},1}$) is $p_{\text{abs},1} = |s_{12}|^2\,\mathcal{E}_{T_0,\omega}^{\text{ther}}\frac{\Delta\omega}{2\pi} + \left(1-|s_{11}|^2-|s_{12}|^2\right)\mathcal{E}_{T,\omega}^{\text{ther}}\frac{\Delta\omega}{2\pi}$. Note that $|s_{12}|^2\,\mathcal{E}_{T_0,\omega}^{\text{ther}}\frac{\Delta\omega}{2\pi}$ is the power collected at port 1 due to the thermal radiation from port 2. The leading coefficient of the second term of $p_{\text{abs},1}$ is found by noting that at thermal equilibrium ($T = T_0$) one needs to

have $p_{\text{abs},1} = p_{\text{rad},1}$. Using $\mathcal{E}_{T,\omega}^{\text{ther}} \approx k_{\text{B}}T$ it follows that the power collected by ports i=1,2 is given by:

$$p_{\text{abs},1} = |s_{12}|^2 k_{\text{B}} T_0 \frac{\Delta\omega}{2\pi} + C_{3\to 1} k_{\text{B}} T \frac{\Delta\omega}{2\pi}. \tag{23a}$$

$$p_{\text{abs},2} = |s_{21}|^2 k_{\text{B}} T_0 \frac{\Delta\omega}{2\pi} + C_{3\to 2} k_{\text{B}} T \frac{\Delta\omega}{2\pi}. \tag{23b}$$

where $C_{3\to 1} = 1 - |s_{11}|^2 - |s_{12}|^2$ and $C_{3\to 2} = 1 - |s_{22}|^2 - |s_{21}|^2$ are the coefficients that determine the coupling between the cavity (index 3) and the ports 1 and 2, respectively. The net power flow $p_i = p_{\text{abs},i} - p_{\text{rad},i}$ at port i can be written as:

$$p_i = C_{3\to i} k_{\text{B}} \left(T - T_0\right) \frac{\Delta\omega}{2\pi}. \tag{24}$$

Thus, $\delta p \equiv p_2 - p_1$ is given by $\delta p = \left(C_{3\to 2} - C_{3\to 1}\right) k_{\text{B}} \left(T - T_0\right) \frac{\Delta\omega}{2\pi}$. This yields the result of the main text with $\Delta C_{\text{D}} = C_{3\to 2} - C_{3\to 1}$.

The directional coupler may be designed such that one of the coefficients $C_{3\to i}$ is negligible in a band-gap. For example, if the directional coupler ensures that when the topological modes rotate in the counter-clockwise direction ($\mathcal{C} < 0$) most of the energy is coupled to port B, then one has $\delta p = -\text{sgn}\left(\mathcal{C}\right) k_{\text{B}} \left(T - T_0\right) |\Delta C_{\text{D}}| \frac{\Delta\omega}{2\pi}$. The coefficients $C_{3\to i}$ are determined by the scattering parameters of the microwave network and hence can be easily determined with a vector network analyzer. In the reciprocal case $s_{21} = s_{12}$ and by symmetry $s_{11} = s_{22}$ so that $\delta p = 0$.

The equivalent noise temperature ($T_{\mathrm{n},i}$) at port i is defined such that $p_{\mathrm{abs},i} = k_B T_{\mathrm{n},i} \frac{\Delta\omega}{2\pi}$ [8]. From the supplementary Eq. (23) it follows that $T_{\mathrm{n},1} = |s_{12}|^2 T_0 + C_{3\to1} T$ and $T_{\mathrm{n},2} = |s_{21}|^2 T_0 + C_{3\to2} T$. If the coupling with the topological cavity is removed, the noise temperature is $T_{\mathrm{n}} = T_0$ (note that the ports 1 and 2 are terminated with matched loads). Hence, the excess of thermal noise in port 2 due to the coupling with the cavity is $\delta T_{\mathrm{n},2} = -\left(1-|s_{21}|^2\right) T_0 + C_{3\to2} T$. For a weak cavity coupling, and a detector with a sufficiently low temperature the first term is negligible ($\left(1-|s_{21}|^2\right) T_0 << T_0$) provided the second term is comparable to or larger than T_0. In these conditions, the excess of noise temperature in the coupled port is roughly $\delta T_{\mathrm{n}} \approx |\Delta C_{\mathrm{D}}| T$.

Supplementary references:

[1] Silveirinha, M. G., Chern Invariants for Continuous Media, *Phys. Rev. B*, **92**, 125153 (2015).

[2] Silveirinha, M. G., Modal expansions in dispersive material systems with application to quantum optics and topological photonics. Preprint at http://arxiv.org/abs/1712.04272.

[3] Agranovich, V., & Ginzburg, V., Spatial Dispersion in Crystal Optics and the Theory of Excitons, (New York: Wiley- Interscience, 1966).

[4] Sakoda, K, Optical Properties of Photonic Crystals, (Springer, Berlin-Heidelberg–New York, 2005).

[5] Silveirinha, M. G., Bulk edge correspondence for topological photonic continua, *Phys. Rev. B*, **94**, 205105 (2016).

[6] Jackson, J. D., Classical Electrodynamics, (Wiley, New York, 1998).

[7] Nyquist, H., Thermal Agitation of Electric Charge in Conductors, *Phys. Rev.* **32**, 110 (1928).

[8] Pozar, D. M., Microwave Engineering, 3rd Ed. (Wiley, Hoboken, NJ, 2005).
